# Spatial variations in the microbiota: comparative analysis of microbial composition and predicted functions across different intestinal segments and feces in donkeys

**DOI:** 10.3389/fmicb.2024.1494926

**Published:** 2025-01-17

**Authors:** Yanwei Wang, Tong Hu, Kaixuan Liang, Shinuo Li, Qiyue Zhang, Wenqiang Li, Honglei Qu, Boying Dong, Haihua Zhang, Qiugang Ma, Ru Jia, Shimeng Huang

**Affiliations:** ^1^Food Processing and Safety, College of Life Sciences, Shanxi University, Taiyuan, China; ^2^National Key Laboratory of Livestock and Poultry Nutrition and Feeding, College of Animal Science and Technology, China Agricultural University, Beijing, China; ^3^Laboratory of Feed Grain Safety and Healthy Poultry Farming, Beijing Jingwa Agricultural Science and Technology Innovation Center, Beijing, China; ^4^College of Animal Science and Veterinary Medicine, Jinzhou Medical University, Jinzhou, Liaoning, China; ^5^National Engineering Research Center for Gelatin-Based Traditional Chinese Medicine, Dong-E-E-Jiao Co., Ltd., Liaocheng, China; ^6^Hebei Key Laboratory of Specialty Animal Germplasm Resources Exploration and Innovation, College of Animal Science and Technology, Hebei Normal University of Science and Technology, Qinhuangdao, China

**Keywords:** donkey, intestine, spatial variations, microbial composition, predicted functions

## Abstract

Donkeys, as single-stomach herbivores, have a complex and diverse microbial community in their digestive tracts. The intestinal bacterial community is crucial for maintaining intestinal homeostasis, as well as the host’s overall nutrition and health. However, research on donkey gut microbes is relatively limited, particularly regarding the microbial colonization patterns in different intestinal segments of adult donkeys. Therefore, this study examined the abundance and function of microbiota across various sites of the intestinal tract (duodenum, jejunum, ileum, cecum, colon) and feces of healthy adult Dezhou male donkeys using 16S rRNA gene sequencing and PICRUSt analysis. The results indicate that donkeys have a rich gut microbial diversity and a large microbial population. No significant differences in the indices of alpha diversity were observed among the donkey’s duodenum, jejunum, ileum, cecum, colon, and feces. A Venn diagram analysis revealed the presence of both unique (Duodenum: 4645; Jejunum: 3586; Ileum: 4904; Cecum: 4253; Colon: 6135; Feces: 4885) and shared (339) ASVs among the different sections. A principal coordinate analysis (PCoA) revealed significant differences (R^2^ = 0.2076, *p* < 0.007) across the six intestinal segments of the donkeys. At the phylum level, Firmicutes (63.64%), Bacteroidetes (20.72%), Verrucomicrobiota (9.16%), Patescibacteria (1.95%), Spirochaetota (1.87%), Actinobacteriota (1.13%), and Proteobacteria (0.42%) were the dominant bacteria in all samples. The Wilcoxon rank-sum test revealed significant differences in the proportions of genera among different intestinal segments. Specific genera were significantly enriched in various segments: *Lachnospiraceae_UCG-008* and *Sphaerochaeta* in the duodenum; *Christensenellaceae_R-7_group* and *Bacillus* in the jejunum; *NK4A214_group* and *Alloprevotella* in the ileum; *UCG-005* and *Lactobacillus* in the cecum; *Clostridium_sensu_stricto_1* and *Chlamydia* in the colon; and *Rikenellaceae_RC9_gut_group* and *Prevotellaceae_UCG-004* in the feces. A PICRUSt2 functional prediction analysis indicated that carbohydrate metabolism, prokaryotic cellular communities, antimicrobial drug resistance, immune diseases, membrane transport, signal transduction, and transcription exhibited significant differences among the different intestinal segments. This study provided critical primary data on the differences in donkey gut microbiota and the synergistic effects between gut microbiota and host functions. These findings can be used to assess donkey health status, improve breeding, and develop microbial additives.

## Introduction

Donkeys, as an important livestock and poultry resource, belong to the Equus family and are typical single-stomached herbivores with multiple valuable attributes (Yang et al., 2023). Donkey products provide significant nutritional benefits, including meat rich in quality protein, essential amino acids, and unsaturated fats. Moreover, their skin is used in the production of Ejiao, a traditional Chinese medicine with various health benefits, while their milk, closely resembling human milk in composition, offers an excellent alternative for dietary needs (Polidori et al., 2015; Camillo et al., 2018; Zhang et al., 2020; Yang et al., 2023). Globally, many different breeds of donkeys exist, among which the Dezhou donkey was considered one of the five most important breeds. The primary breeding goal for Dezhou donkeys is the production of high-quality fur and meat (Seyiti and Kelimu, 2021). Furthermore, there has been an increasing emphasis on boosting fur and meat production in this breed (Liu et al., 2022; Wang et al., 2022). Given donkeys’ substantial medicinal and nutritional value, the global community is paying increasing attention to the development of donkey animal husbandry. Consequently, to achieve higher yields and meet the growing market demand for donkey-related products, it has become necessary to prioritize these animals’ growth, development, and health based on their unique digestive mechanism.

The interplay between the commensal microbiota and mammalian growth, development, and immune system function involves multifaceted interactions in both homeostasis and disease (Eckburg et al., 2005; Durack and Lynch, 2019; Zheng et al., 2020; Hou et al., 2022). Numerous studies have demonstrated that the intestinal microbiome is directly or indirectly involved in host metabolism, physiology, and immunity through alterations in microbial population structure, metabolite production, signal transduction, and gene expression (Li et al., 2019; Hou et al., 2022). These changes contribute to the formation of complex and mutually adaptive micro-ecological systems, with the stable microbiome–host homeostasis being essential for maintaining optimal physiological function of the intestine (Ley et al., 2008; Zhang et al., 2020). The metabolites produced by gut microbes are highly diverse, including short-chain fatty acids such as acetate, propionate, and butyrate, as well as alcohol, carbon dioxide, and hydrogen (Brestoff and Artis, 2013). For example, *Faecalibacterium prausnitzii* and *Eubacterium rectale* produce butyrate, which helps reduce mild inflammation, glucose metabolism imbalances, and insulin resistance in the host (Jia et al., 2021). Additionally, the complex and diverse gut microbes metabolize carbohydrates directly (Liu et al., 2014; Hou et al., 2022). Therefore, understanding the intestinal microbial composition of donkeys could aid in better regulating their production performance and improving their health. However, there are limited studies reporting on the microbiota in the intestinal tracts of healthy donkeys.

In the present study, which aimed to determine the baseline state of microbiota across different intestinal segments in donkeys, we conducted a systematic survey of the duodenum, jejunum, ileum, cecum, colon, and feces from six healthy Dezhou male donkeys. This comprehensive analysis provided insights into the microbial communities present in each segment, laying the foundation for understanding the role of the intestinal microbiome in donkey health and production performance.

## Materials and methods

### Animal ethics statement

The utilization of animals in this study adhered to rigorous ethical standards and was formally approved by the Animal Care and Use Committee of China Agricultural University (Approval No: AW81704202-1-1).

### Experimental design

Six healthy Dezhou male donkeys (age: 2 to 2.5 years; body weight: 250 ± 10 kg) with similar birth times and weights were selected for this study. All donkeys were raised under the same farming conditions at Shandong Dong-E–E-Jiao Co., Ltd.^1^ Table 1 shows the ingredients and nutritional content of the concentrates, which were provided according to the farm’s program. Additionally, roughage was provided as cereal straw available *ad libitum*, with free access to water. The donkeys were fed twice daily at 07:00 and 19:00. During the study period, the Dezhou male donkeys were not fed probiotics or antibiotics for at least three months. They were confirmed as healthy by a veterinarian and did not suffer from intestinal diseases.

**Table 1:** Composition and nutrient levels of basal and experimental diet (air-dry basis %).

Items	Ingredient (%)
Ingredients	
Corn	30.00
Soybean meal	21.00
Rice bran meal	14.00
Wheat	12.00
Wheat middling and red dog	11.80
Limestone	4.00
Dicalcium phosphate	1.60
Salt	0.60
Zeolite meal	3.00
Premix^1^	2.00
Total	100.00
Nutritional value analysis^2^	
Dry matter (%)	88.22
Digestible energy, Mcal/kg	2.81
Crude fat (%)	2.18
Total Ca (%)	1.98
Total P (%)	0.84
Crude protein (%)	17.46
Neutral detergent fiber (%)	10.99
Acid detergent fiber (%)	4.01

^1^The premix provided the following per kg of diets: vitamin A 12,000 IU, vitamin D_3_ 1,500 IU, vitamin E 40.0 mg, vitamin K_3_ 3.0 mg, vitamin B_1_ 2.0 mg, vitamin B_2_ 4.0 mg, vitamin B_6_ 4.0 mg, vitamin B_12_ 10.0 μg, pantothenic acid 20.0 mg, niacin 30.0 mg, folacin 1.7 mg, biotin 0.3 mg, Fe (as FeSO_4_) 150.0 mg, Cu (as CuSO_4_) 40.0 mg, Mn (as MnO) 100.0 mg, Se (as Na_2_SeO_3_) 0.4 mg, I (as KI) 0.6 mg, Zn (as ZnSO_4_) 220.0 mg. ^2^Calculated values.

The donkeys underwent a 12-h feed withdrawal before slaughter. The six donkeys were stunned by an electrical stunning device and then slaughtered at Dong-E–E-Jiao Co., Ltd. (Zhang et al., 2022). The organs of the intestinal tract were carefully separated and removed. Contents samples were collected from different intestinal segments (duodenum, jejunum, ileum, cecum, and colon) of each donkey as soon as possible. Stool samples were collected near the anus. All contents were collected, handled, and stored aseptically to prevent contamination. The samples were packed into 2 mL centrifuge tubes, quickly frozen in liquid nitrogen, and then stored on dry ice for transportation to the laboratory, where they were stored at −80°C for further analysis.

### Microbiota sequencing and analysis

Total DNA was extracted from 200 mg of each fecal specimen using the E.Z.N.A.® Soil DNA Kit (Omega Bio-tek, Norcross, GA, USA) according to the manufacturer’s protocol (*n* = six samples per group). The extracted DNA was used as a template to amplify the V3-V4 hypervariable region of the bacterial 16S rRNA gene using universal primers 338F (5′-ACTCCTACGGGAGGCAGCA-3′) and 806R (5′-GGACTACHVGGGTWTCTAAT-3′). The amplified products were detected with agarose gel electrophoresis (2% agarose), recovered using the AxyPrep DNA Gel Recovery Kit (Axygen Biosciences, Union City, CA, USA), and quantified using a Qubit 2.0 Fluorometer (Thermo Fisher Scientific, Waltham, MA, USA) to pool into equimolar amounts. Amplicon libraries were sequenced on the Illumina HiSeq 2,500 platform (Illumina, San Diego, CA, USA) for paired-end reads of 250 bp. The amplicons were purified from agarose gels using the AxyPrep DNA Gel Extraction Kit (Corning, Glendale, USA), pooled in equimolar amounts, and sequenced on an Illumina MiSeq platform (Illumina, San Diego, USA) following the standard protocol of Majorbio Bio-Pharm Technology Co., Ltd. (Shanghai, China).

Illumina sequencing data were filtered, denoised, concatenated, and de-embedded using QIIME2 to obtain high-quality sequencing data, with each sample region yielding ≥50,000 effective sequences for subsequent bioinformatics analysis. Tags were clustered into ASVs using DADA2. ASV taxonomic assignments were conducted using the RDP classifier (version 2.2) and annotated in the Silva1 database. Alpha diversity indices, including ACE, Chao, Shannon, and Sobs, were calculated using QIIME2 and the R package vegan (v2.5.6). A Venn diagram showing the number of shared and unique ASVs among the different intestinal segments was constructed using the VennDiagram package in R (v3.1.1). Principal coordinate analysis (PCoA) and permutational multivariate analysis of variance (Adonis) were carried out using R software (version 3.2.1).^2^ Genus-level microbial differences between different intestinal segments were analyzed using vegan v3.5.1, with comparisons conducted using the Wilcoxon rank-sum test or Kruskal–Wallis rank sum test and pairwise comparisons to identify specific variations. Additionally, PICRUSt2 (v2.5.0)^3^ was used to predict metagenome functions based on the Kyoto Encyclopedia of Genes and Genomes (KEGG), addressing different aspects of functionality and ecological significance. This enriched the analysis and interpretations of microbial community functional metabolic activities based on marker gene sequencing profiles using the KEGG Orthology database.

### Statistical analysis

All statistical analyses were conducted using GraphPad Prism (version 9.0; GraphPad Software, La Jolla, CA, USA). Differences were evaluated with groupwise comparisons using Student’s *t*-test, the Mann–Whitney test, and one- or two-way ANOVA corrected for multiple comparisons with *post hoc* Tukey tests. Data are shown as mean values ± standard errors of the mean (SEMs). *p* < 0.05 were considered statistically significant.

## Results

### Analysis of alpha diversity across all samples

**Figure 1:**
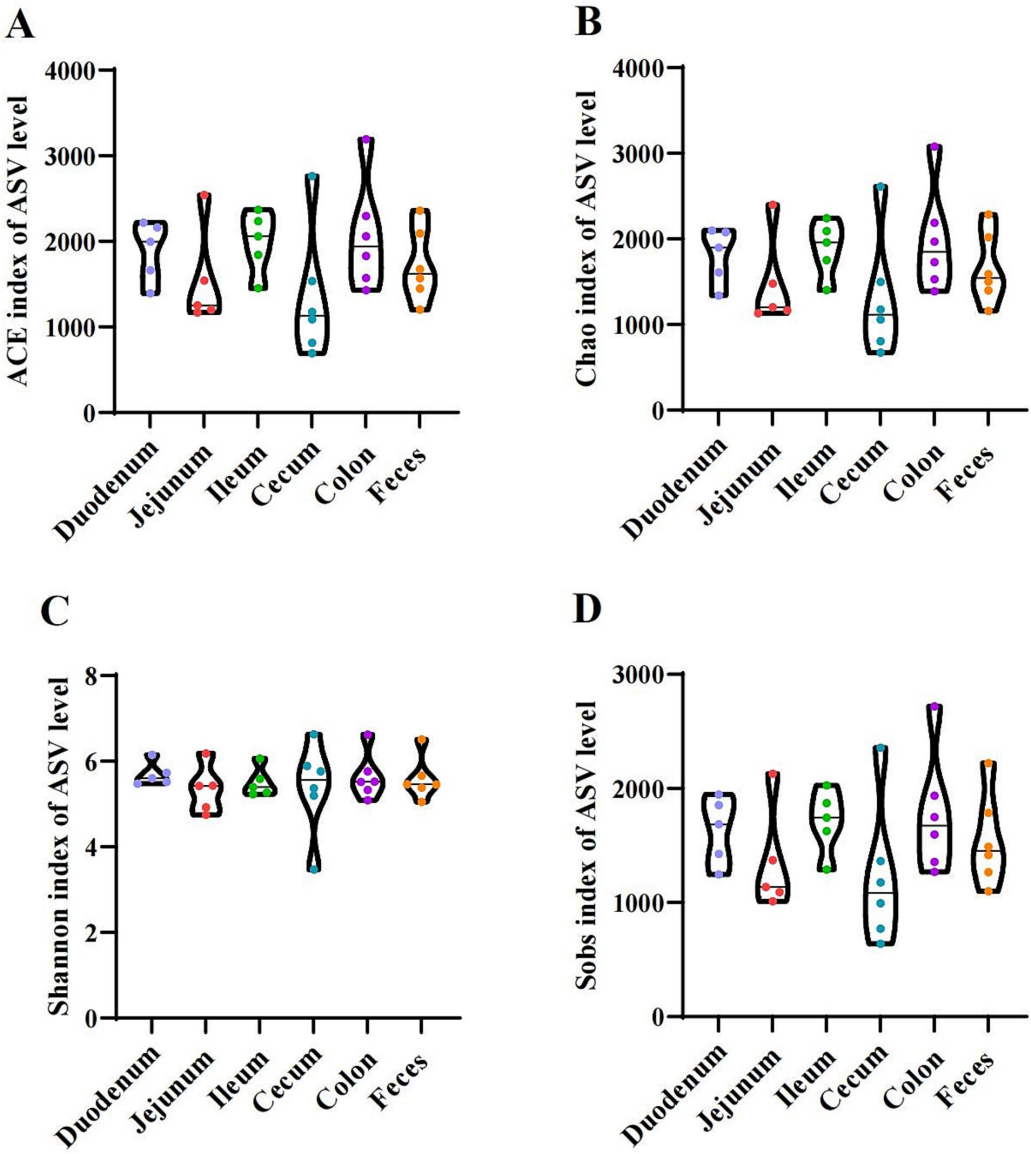
Alpha diversity indices of the duodenum, jejunum, ileum, cecum, colon, and feces in donkeys. **(A)** Ace index. **(B)** Chao index. **(C)** Shannon index. **(D)** Sobs index.

As shown in Figure 1, the indices of alpha diversity include the Ace (Figure 1A), Chao (Figure 1B), Shannon (Figure 1C), and Sobs (Figure 1D) indices. No significant differences were observed among the donkey’s duodenum, jejunum, ileum, cecum, colon, and feces (*p* > 0.05).

### Overall microbiota profile and spatial variation

**Figure 2:**
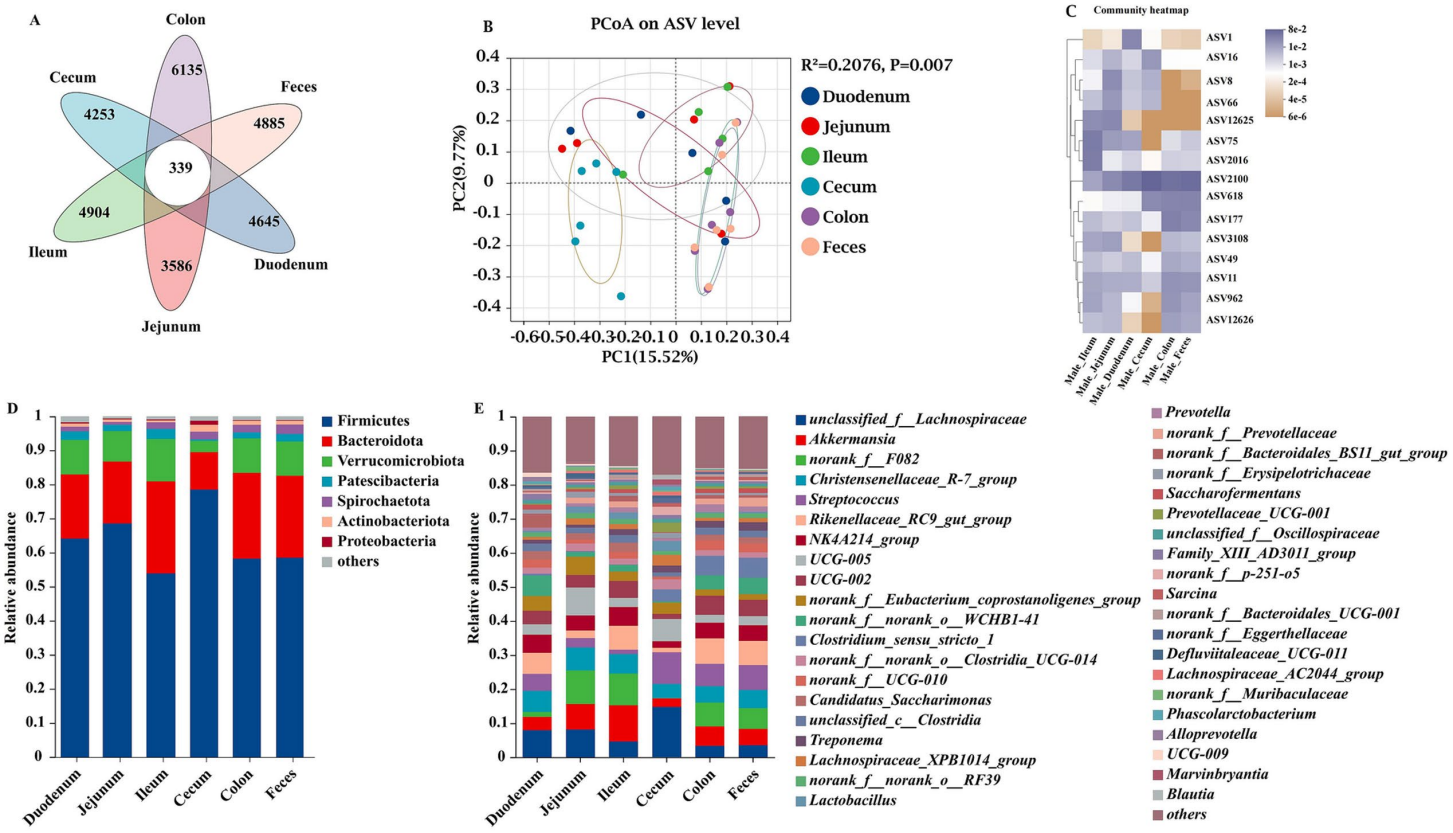
Unique bacterial composition of intestinal microbiota between groups at different sites of donkeys. Venn diagram, where areas of overlap indicate the numbers of ASVs shared among the overlapping groups. A principal coordinate analysis (PCoA) was performed to examine the relative abundance of microbial species, with Spearman’s coefficient distance. The colored dots represented different intestinal segments and feces samples, and the colors indicated different cohorts. The x-axis is principal coordinate 1 (PC1), and the y-axis is principal coordinate 2 (PC2). **(A)** Overall comparison of Venn diagram. **(B)** Overall comparison of PCoA. **(C)** The top 15 species of ASV. Taxonomic composition at the phylum **(D)** and genus **(E)** levels.

The microbial species from the intestinal contents (duodenum, jejunum, ileum, cecum, and colon) and fecal samples showed high richness and diversity in the present study (Figure 2). To illustrate the distribution of both the shared and unique ASVs among the samples, we employed a Venn diagram to depict the distribution of bacterial community ASVs. These six groups shared a community containing 339 ASVs (Figure 2A). The duodenum, jejunum, ileum, cecum, colon, and feces contained 4,645, 3,586, 4,904, 4,253, 6,135, and 4,885 unique ASVs, respectively (Figure 2A). At the ASV level, principal coordinate analysis (PCoA) of *β*-diversity, assessed using Bray–Curtis dissimilarity, was used to evaluate sample community similarity. Our findings revealed a significant separation among the six groups (*p* < 0.05) (Figure 2B). The top 15 species (ASV1, ASV16, ASV8, ASV66, ASV12625, ASV75, ASV2016, ASV2100, ASV618, ASV177, ASV3108, ASV49, ASV11, ASV962, and ASV12626) with the highest abundance were selected at the ASV level (Figure 2C). The top seven bacterial phyla of the gut microbiota were Firmicutes, Bacteroidota, Verrucomicrobiota, Patescibacteria, Spirochaetota, Actinobacteriota, and Proteobacteria (Figure 2D). *Akkermansia*, *Christensenellaceae_R-7_group*, *Streptococcus*, *Rikenellaceae_RC9_gut_group*, *NK4A214_group*, *UCG-005*, *UCG-002*, *Clostridium_sensu_stricto_1*, *Candidatus_Saccharimonas*, *Treponema*, *Lachnospiraceae_XPB1014_group*, and *Lactobacillus* were the predominantly abundant genera (Figure 2E).

### Comparative analysis of duodenal and other intestinal segments

**Figure 3:**
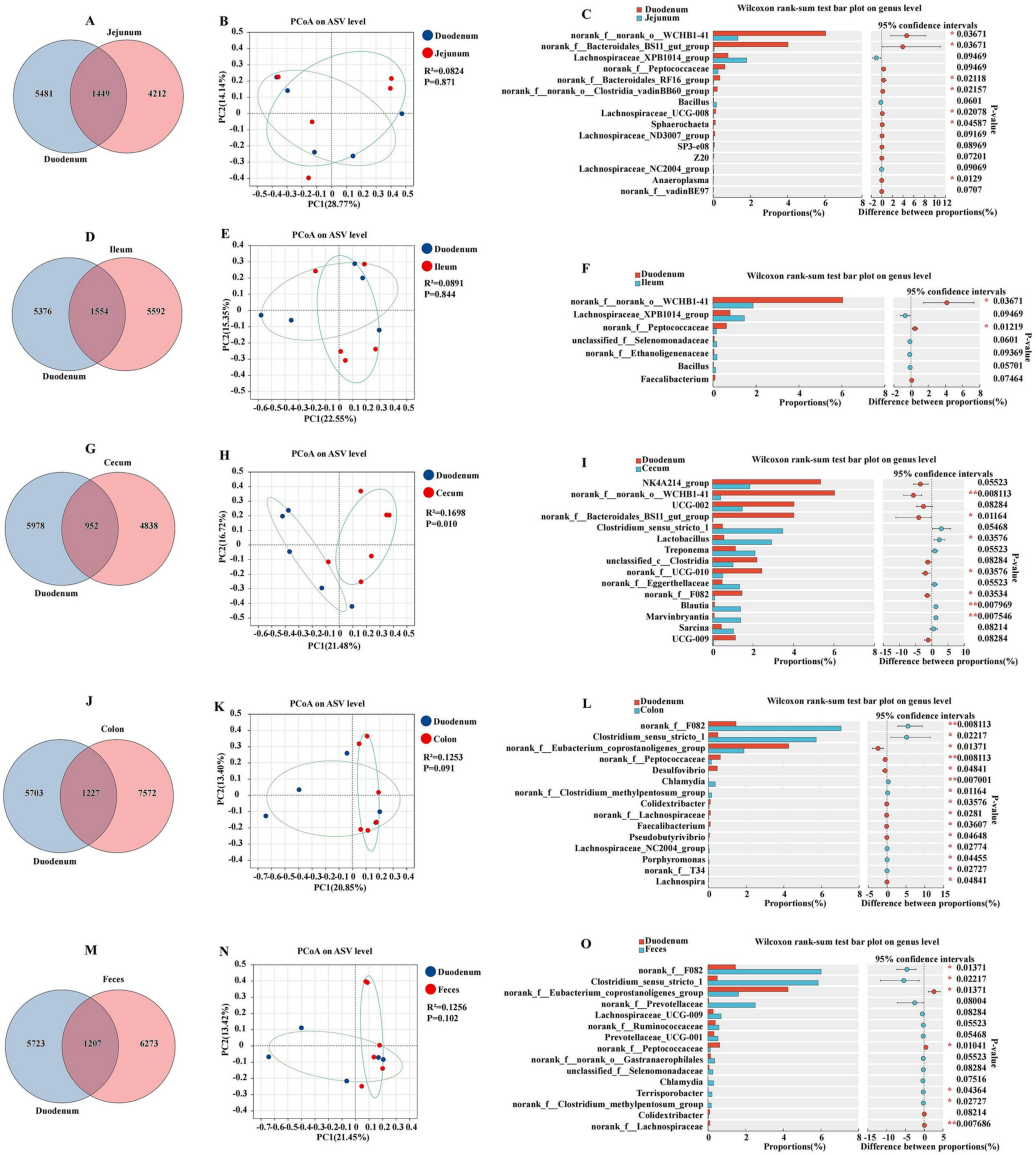
Differences between the duodenum, other intestinal segments, and feces were analyzed using various methods. The Venn diagram shows the amplicon sequence variant (ASV) distribution of bacterial communities in donkeys, indicating differences between the duodenum and jejunum **(A)**, ileum **(D)**, cecum **(G)**, colon **(J)**, and feces **(M)**. A principal coordinate analysis (PCoA) was performed to display the relative abundance of microbial species, with Spearman’s coefficient distance, between the duodenum and jejunum **(B)**, ileum **(E)**, cecum **(H)**, colon **(K)**, and feces **(N)**. The Wilcoxon rank sum test bar plot at the genus level reveals distinct species in the duodenum and jejunum **(C)**, ileum **(F)**, cecum **(I)**, colon **(L)**, and feces **(O)**. *p ≤ 0.05, *p* ≤ 0.01.

The comparative analysis of the microbiota across different intestinal segments revealed distinct characteristics of the duodenal microbiome when compared to the jejunum, ileum, cecum, colon, and feces. The results were presented in a series of Venn diagrams, PCoA plots, and bar charts to elucidate the variations in microbial composition and diversity (Figure 3). The duodenum and jejunum had a total of 1,449 ASVs, with 5,481 and 4,212 ASVs, respectively (Figure 3A). The PCoA plot of ASV showed a moderate separation between the duodenum and jejunum, with a coefficient of determination R^2^ of 0.0824 and a *p*-value of 0.871, suggesting that while differences existed, they did not strongly cluster the samples (Figure 3B). The Wilcoxon rank sum test indicated significant differences in the relative abundance of several bacterial genera between the duodenum and jejunum (*p* < 0.05). Notably, the genera *Anaeroplasma*, *Sphaerochaeta*, and *Lachnospiraceae_UCG-008* were found in higher proportions in the duodenum, while the *Lachnospiraceae_NC2004_group* and *Bacillus* were more abundant in the jejunum (Figure 3C). Similar to the jejunum, the ileum also exhibited a distinct microbiota composition compared to the duodenum. The duodenum and ileum had a total of 1,554 ASVs, with 5,376 and 5,592 ASVs, respectively, (Figure 3D). The PCoA plot for the duodenum and ileum had an R^2^ of 0.0891 and a *p*-value of 0.844, indicating a moderate level of separation between these two segments (Figure 3E). Genera such as *norank_f__norank_o__WCHB1-41* and *norank_f__Peptococcaceae* were differentially abundant, with *p*-values indicating significant differences (*p* < 0.05) (Figure 3F). The duodenum and cecum had a total of 952 ASVs, with 5,978 and 4,838 ASVs, respectively, (Figure 3G). The PCoA plot revealed a stronger separation between the duodenum and cecum, with an R^2^ of 0.1698 and a *p*-value of 0.010, suggesting a more pronounced difference in microbial composition (Figure 3H). The duodenum and cecum showed a significant divergence in their microbial communities, with the cecum having a higher relative abundance of certain genera such as *Lactobacillus*, *Blautia*, and *Marvinbryantia* (*p* < 0.05) (Figure 3I). The duodenum and colon had a total of 1,227 ASVs, with 5,703 and 7,572 ASVs, respectively, (Figure 3J). The PCoA plot had an R^2^ of 0.1253 and a *p*-value of 0.091, indicating a moderate level of clustering based on differences in the microbiota (Figure 3K). A comparison between the duodenum and colon revealed a moderate level of difference in microbial composition, with certain genera such as *Clostridium_sensu_stricto_1*, *Desulfovibrio*, *Chlamydia*, *Colidextribacter*, *Faecalibacterium, Pseudobutyrivibrio*, *Lachnospiraceae_NC2004_group*, *Porphyromonas*, and *Lachnospira* showing significant variation between the two segments (Figure 3L). The duodenum and feces had a total of 1,207 ASVs, with 5,723 and 6,273 ASVs, respectively, (Figure 3M). The PCoA plot for this comparison had an R^2^ of 0.1256 and a *p*-value of 0.102, suggesting some degree of separation in the microbial communities (Figure 3N). The duodenum and feces microbiota also displayed differences, with the feces having a higher abundance of genera such as *Clostridium_sensu_stricto_1* and *Terrisporobacter* (Figure 3O).

### Comparative analysis of jejunal and other intestinal segments

**Figure 4:**
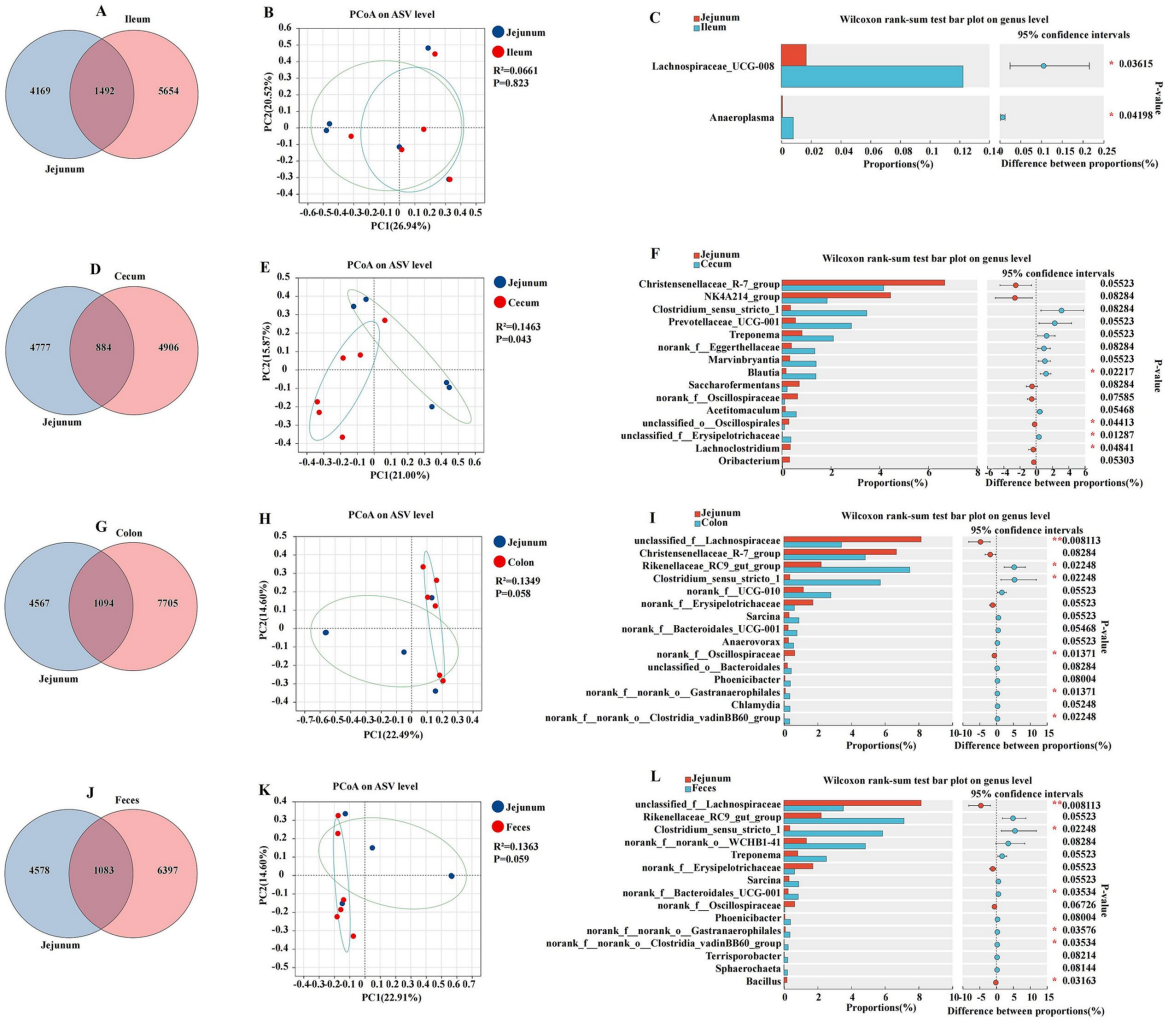
Differences between the jejunum and other intestinal segments and feces were analyzed using various methods. The Venn diagram shows the amplicon sequence variant (ASV) distribution of bacterial communities in donkeys, indicating differences between the jejunum and ileum **(A)**, cecum **(D)**, colon **(G)**, and feces **(J)**. A principal coordinate analysis (PCoA) was performed to depict the relative abundance of microbial species, with Spearman’s coefficient distance, between the jejunum and ileum **(B)**, cecum **(E)**, colon **(H)**, and feces **(K)**. The Wilcoxon rank sum test bar plot at the genus level highlights the distinct species in the jejunum and ileum **(C)**, cecum **(F)**, colon **(I)**, and feces **(L)**. *p ≤ 0.05, **p ≤ 0.01.

To elucidate the microbial diversity and community structure within the jejunum and its comparison to other intestinal segments, including the ileum, cecum, colon, and feces, we analyzed the Venn diagram, PCoA, and amplicon sequencing data at the genus level to assess the microbial community composition and diversity (Figure 4). The jejunum and ileum were characterized by a total abundance of 1,492 and individual counts of 4,169 and 5,654 ASVs, respectively (Figure 4A). The PCoA plot of ASV showed a moderate separation between the jejunum and ileum, with a coefficient of determination R^2^ of 0.0661 and a *p*-value of 0.823 (Figure 4B). The Wilcoxon rank sum test indicated significant differences in the relative abundance of certain bacterial genera between the jejunum and ileum. Notable genera such as *Anaeroplasma* and *Lachnospiraceae_UCG-008* were differentially abundant, with *p*-values indicating significant differences (*p* < 0.05) (Figure 4C). The jejunum and cecum were characterized by a total abundance of 884 and individual counts of 4,777 and 4,906 ASVs, respectively (Figure 4D). The comparison between the jejunum and cecum revealed a more pronounced divergence in their microbial communities. The PCoA plot had an R^2^ of 0.1463 and a *p*-value of 0.043, suggesting a significant separation between these two segments (Figure 4E). Genera such as *unclassified_o__Oscillospirales* and *Lachnoclostridium* were found in higher proportions in the jejunum, while others such as *Blautia* and *unclassified_f__Erysipelotrichaceae* were more abundant in the cecum (*p* < 0.05) (Figure 4F). The jejunum and colon were characterized by a total abundance of 1,094 and individual counts of 4,567 and 7,705 ASVs, respectively (Figure 4G). The jejunum and colon also exhibited distinct microbial compositions. The PCoA plot for this comparison had an R^2^ of 0.1349 and a *p*-value of 0.058, indicating a moderate level of separation (Figure 4H). The Wilcoxon rank-sum test revealed significant differences in the proportions of genera such as *unclassified_f__Lachnospiraceae* and *norank_f__Oscillospiraceae*, with the jejunum showing a higher abundance of these genera compared to the colon (*p* < 0.05) (Figure 4I). The jejunum and feces were characterized by a total abundance of 1,083 and individual counts of 4,578 and 6,397 ASVs, respectively (Figure 4J). The jejunum and feces microbiota displayed differences in their microbial community structure. The PCoA plot had an R^2^ of 0.1363 and a *p*-value of 0.059, suggesting some degree of separation in the microbial communities (Figure 4K). Genera such as *unclassified_f__Lachnospiraceae* and *Bacillus* were differentially abundant between the jejunum and feces, with the jejunum having a higher relative abundance of these genera (*p* < 0.05) (Figure 4L).

### Comparative analysis of ileal and other intestinal segments

**Figure 5:**
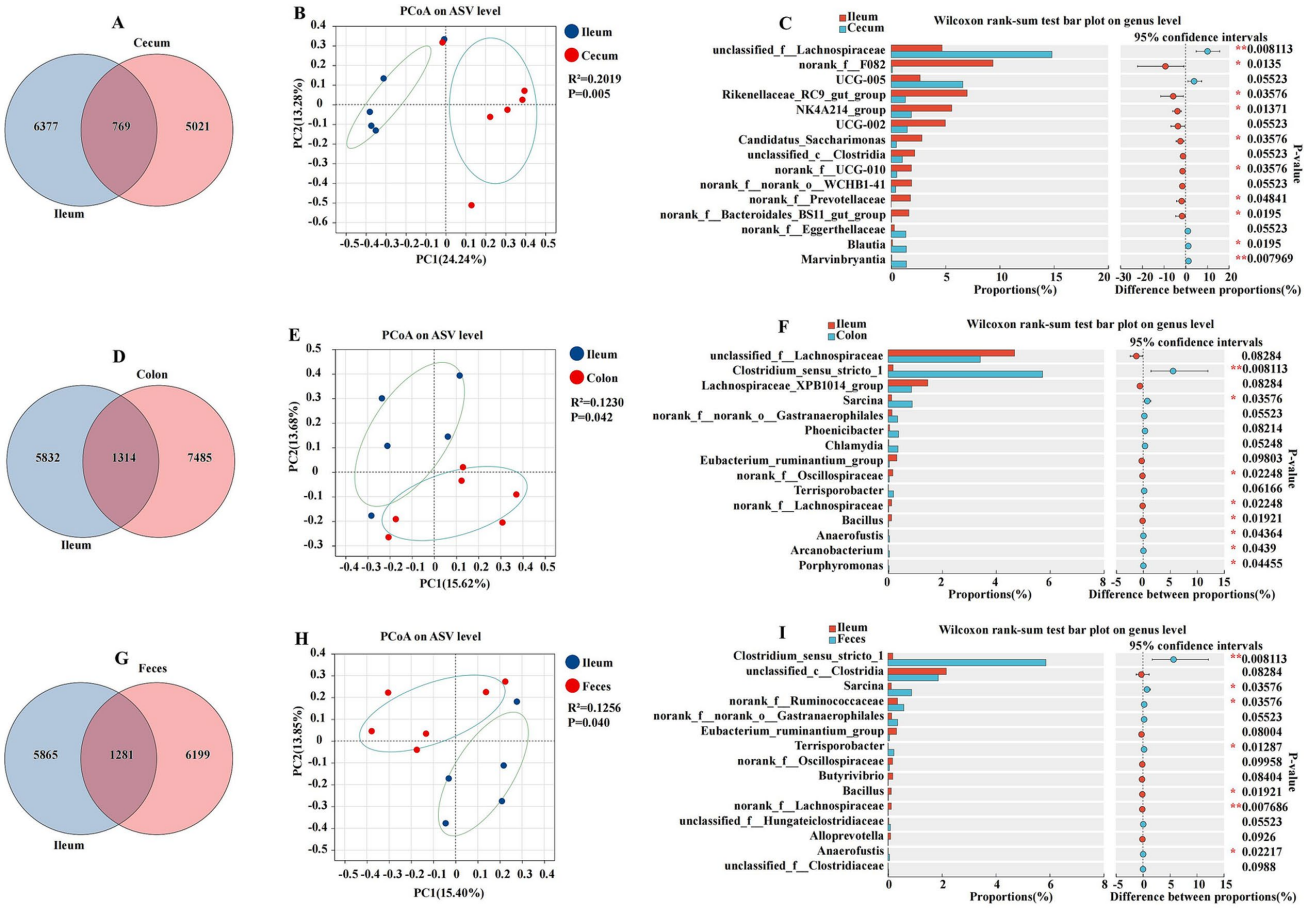
Differences between the ileum and other intestinal segments and feces analyzed using various methods. The Venn diagram shows the amplicon sequence variant (ASV) distribution of bacterial communities in donkeys, indicating differences between the ileum and cecum **(A)**, colon **(D)**, and feces **(G)**. A principal coordinate analysis (PCoA) was performed to examine displayed the relative abundance of microbial species with Spearman’s coefficient distance, between the ileum and cecum **(B)**, colon **(E)**, and feces **(H)**. The Wilcoxon rank-sum test bar plot at the genus level depicts the distinct species in the ileum and cecum **(C)**, colon **(F)**, and feces **(I)**. *p ≤ 0.05, **p ≤ 0.01.

This section presents a comparative analysis of the ileal microbiota with other intestinal segments, including the cecum, colon, and feces. This study utilized amplicon sequencing to identify and quantify the microbial community at the genus level, and PCoA was employed to visualize the differences in microbial composition (Figure 5). The ileum and cecum were characterized by total abundances of 769 and individual counts of 6,377 and 5,021 ASVs, respectively (Figure 5A). The PCoA plot of the abundance of ASVs revealed a moderate separation between the ileum and cecum, with a coefficient of determination (R^2^) of 0.1230 and a *p*-value of 0.042 (Figure 5B). The Wilcoxon rank sum test indicated significant differences in the relative abundance of several bacterial genera between the ileum and cecum. Genera such as *Rikenellaceae_RC9_gut_group*, *NK4A214_group*, *Candidatus_Saccharimonas*, *norank_f__Prevotellaceae*, and *norank_f__Bacteroidales_BS11_gut_group* were found in higher proportions in the ileum, while *unclassified_f__Lachnospiraceae*, *Blautia*, and *Marvinbryantia* were more abundant in the cecum (*p* < 0.05) (Figure 5C). The ileum and colon were characterized by total abundances of 1,314 and individual counts of 5,832 and 7,485 ASVs, respectively (Figure 5D). The ileum and colon also exhibited distinct microbial compositions. The PCoA plot had an R^2^ of 0.2019 and a *p*-value of 0.005, indicating a significant separation between these two segments (Figure 5E). The Wilcoxon rank-sum test bar plot showed significant differences in the proportions of genera such as *norank_f__Oscillospiraceae*, *norank_f__Lachnospiraceae*, and *Bacillus*, with the ileum having a higher relative abundance of these genera compared to the colon (*p* < 0.05) (Figure 5F). The ileum and feces were characterized by a total abundance of 1,281 and individual counts of 5,865 and 6,199 ASVs, respectively (Figure 5G). The PCoA plot had an R^2^ of 0.1256 and a *p*-value of 0.040, suggesting some degree of separation in the microbial communities (Figure 5H). Genera such as *Bacillus* and *norank_f__Lachnospiraceae* were differentially abundant between the ileum and feces, with the ileum showing a higher abundance of these genera (*p* < 0.05) (Figure 5I).

### Comparative analysis of cecal and other intestinal segments

**Figure 6:**
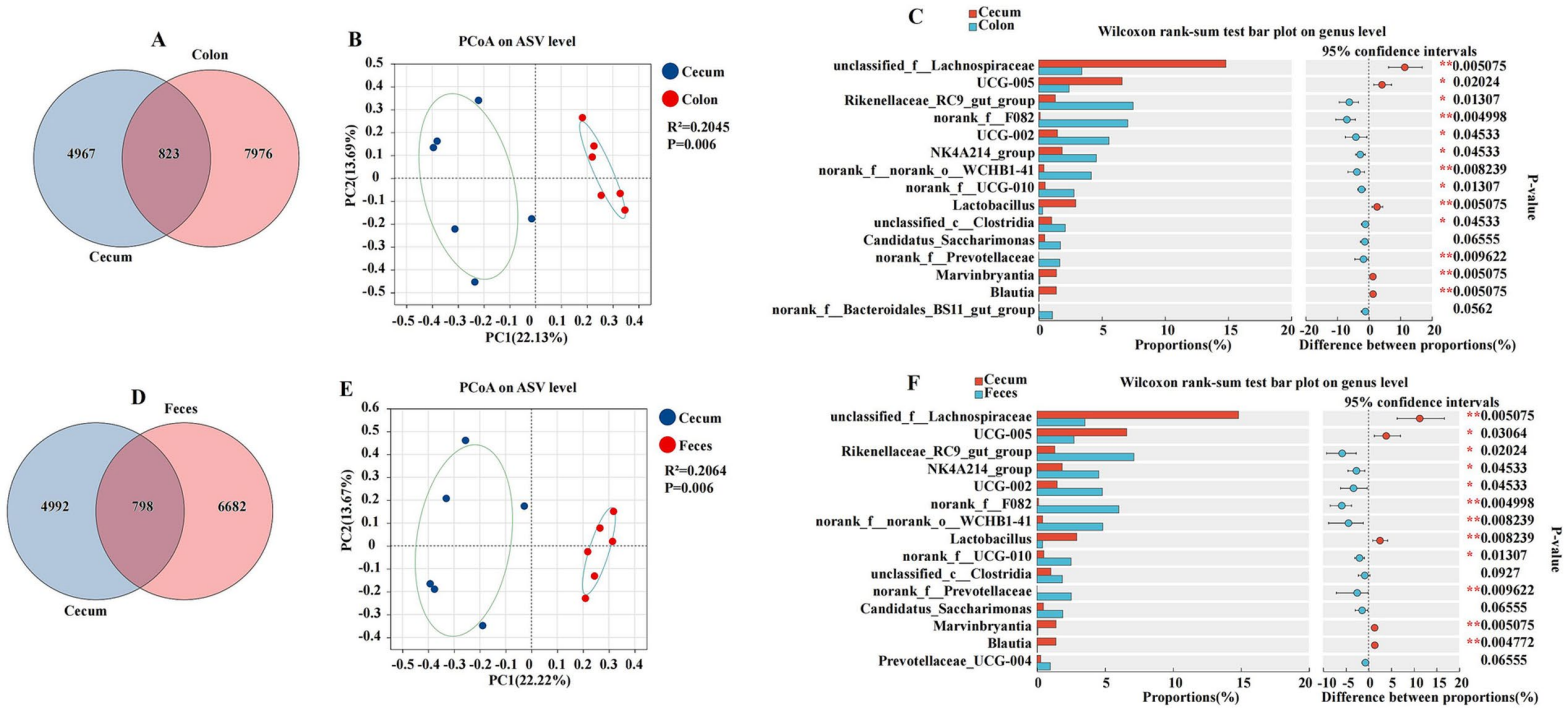
Differences between the cecum, colon, and feces were analyzed using different methods. The Venn diagram shows the amplicon sequence variant (ASV) distribution of bacterial communities in donkeys, indicating differences between the cecum and colon **(A)**, and feces **(D)**. A principal coordinate analysis (PCOA) was performed to show the relative abundance of microbial species, with Spearman’s coefficient distance, between the cecum and colon **(B)**, and feces **(E)**. The Wilcoxon rank sum test bar plot at the genus level shows the distinct species in the cecum and colon **(C)**, and feces **(F)**. *p ≤ 0.05, *p* ≤ 0.01.

This section details the comparative analysis of the cecal microbiota with those of the colon and feces, providing insights into the unique microbial characteristics of the cecum within the gastrointestinal tract (Figure 6). The cecum and colon were characterized by a total abundance of 823 and individual counts of 4,967 and 7,976 ASVs, respectively (Figure 6A). The PCoA plot revealed a significant separation between the cecum and colon, with a coefficient of determination R2 of 0.2045 and a *p*-value of 0.006, indicating a substantial difference in microbial composition (Figure 6B). Genera such as *unclassified_f__Lachnospiraceae*, *UCG-005*, *Lactobacillus*, *Marvinbryantia*, and *Blautia* were found in higher proportions in the cecum, while other genera like *Rikenellaceae_RC9_gut_group*, *norank_f__F082*, *UCG-002*, *NK4A214_group*, *norank_f__norank_o__WCHB1-41*, *norank_f__UCG-010*, and *unclassified_c__Clostridia*, *norank_f__Prevotellaceae* were more abundant in the colon (*p* < 0.05) (Figure 6C). The cecum and feces were characterized by a total abundance of 798 and individual counts of 4,992 and 6,682 ASVs, respectively (Figure 6D). The comparison between the cecum and feces microbiota also showed distinct differences. The PCoA plot had an R2 of 0.2064 and a *p*-value of 0.006, suggesting a significant divergence in microbial composition (Figure 6E). Genera such as *unclassified_f__Lachnospiraceae*, *UCG-005*, and *Lactobacillus* were more prevalent in the cecum, whereas *Rikenellaceae_RC9_gut_group*, *NK4A214_group*, *UCG-002*, *norank_f__F082*, *norank_f__norank_o__WCHB1-41*, *norank_f__UCG-010*, and *norank_f__Prevotellaceae* were found in higher proportions in the feces (*p* < 0.05) (Figure 6F).

### Comparative analysis of colonic and fecal segments

**Figure 7:**
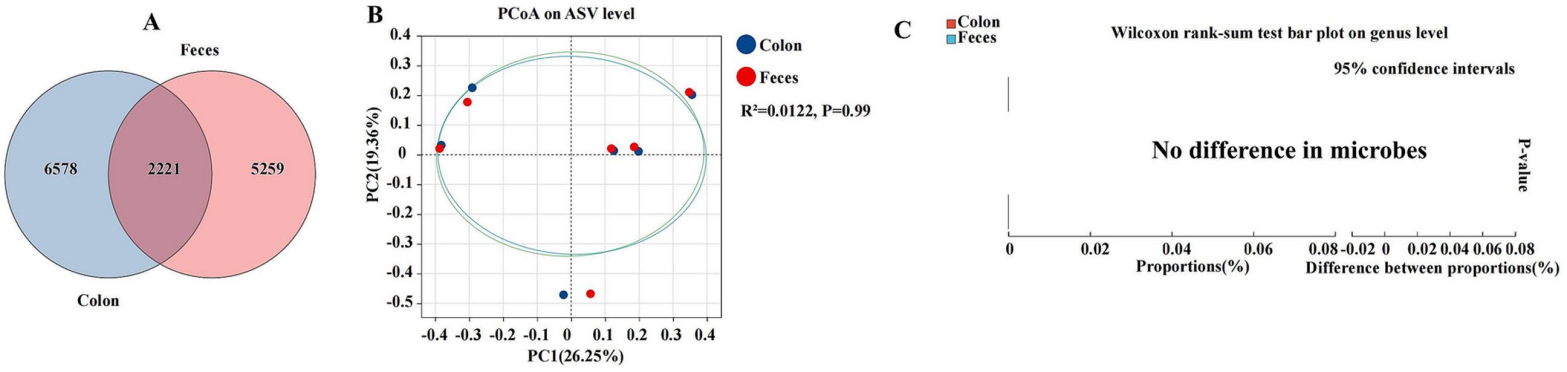
Differences between colon and feces were analyzed using different methods. The Venn diagram shows the amplicon sequence variant (ASV) distribution of bacterial communities in colon and feces of donkeys **(A)**. A principal coordinate analysis (PCOA) was performed to show the relative abundance of microbial species with Spearman’s coefficient distance, in the colon and feces **(B)**. The Wilcoxon rank sum test bar plot at the genus level shows the distinct species in the cecum and feces **(C)**. *p ≤ 0.05, **p ≤ 0.01.

We characterized and explored the differences in the gut microbiota between the colon and feces, two segments of the gastrointestinal tract that are pivotal in digestion and host microbial interactions (Figure 7). The colon and feces were characterized by a total abundance of 2,221 and individual counts of 6,578 and 5,259 ASVs, respectively (Figure 7A). The PCoA plot revealed a low coefficient of determination (R^2^) of 0.0122, with a non-significant *p*-value of 0.99, indicating minimal separation between the colon and feces microbial communities (Figure 7B). The Wilcoxon rank-sum test, a non-parametric statistical test used to compare the distributions of two samples, showed no significant differences in the relative abundance of microbial genera between the colon and feces. The bar plot of proportions and the difference between proportions did not reveal any substantial variations, suggesting a high degree of similarity in microbial composition (Figure 7C).

### Variation in the anticipated microbial genetic functionalities spanning the entire cohort of specimens

**Figure 8:**
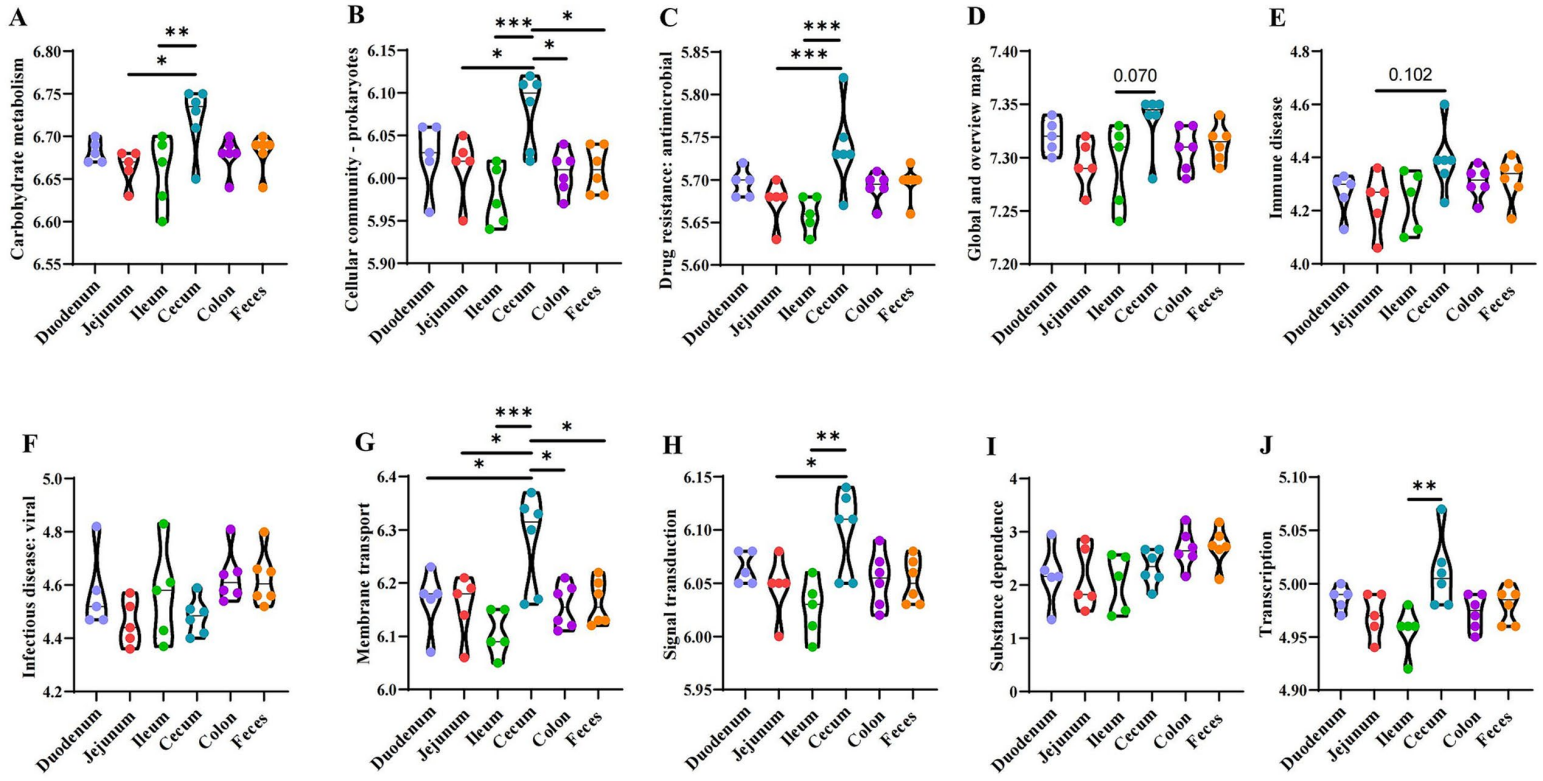
Analysis of the functional genes of microbiota. **(A)** Carbohydrate metabolism. **(B)** Cellular community - prokaryotes. **(C)** Drug resistance: antimicrobial. **(D)** Global and overview maps. **(E)** Immune disease. **(F)** Infectious disease: viral. **(G)** Membrane transport. **(H)** Signal transduction. **(I)** Substance dependence. **(J)** Transcription. *p ≤ 0.05, **p ≤ 0.01, ***p ≤ 0.001.

Based on the Kyoto Encyclopedia of Genes and Genomes (KEGG) metabolic pathways composition and differences analysis, the differences and changes in functional genes in microbial communities between samples from different sites were observed, which allowed the study of community samples’ metabolic function changes in response to environmental changes (Figure 8). The functional prediction revealed a significant variance in carbohydrate metabolism, with the highest scores observed in the cecum (*p* < 0.05) (Figure 8A), indicating a potentially rich capacity for the fermentation of complex carbohydrates in these segments. The analysis highlighted a diverse range of prokaryotic functions, with the cecum showing the highest predicted functionality (*p* < 0.05) (Figure 8B), suggesting a more complex microbial interaction and community structure in these regions. A high level of predicted drug resistance genes was observed, with the highest scores in the cecum (*p* < 0.05) (Figure 8C), which may have implications for the spread of antimicrobial resistance in the gut ecosystem. The overall functional maps indicated a broad metabolic and genetic potential across all samples, with subtle variations that might reflect the adaptability of the microbiota to different intestinal environments (Figure 8D). The predictive function of cecum species related to immune diseases showed a higher tendency, suggesting a potential role of the gut microbiota in modulating local immune responses (Figure 8E). There were no significant differences among the predicted capacities for responding to viral infections, which could be linked to their strategic roles in pathogen defense and antigen sampling (Figure 8F). Membrane transport functions were predicted to be consistently present across all samples, with the cecum showing a slightly higher capacity, which is crucial for nutrient uptake and ion balance (Figure 8G). Signal transduction mechanisms were predicted to be variably present, with the highest functionality in the cecum, indicating a complex communication network within the gut microbiota (Figure 8H). The predicted functions related to substance dependence were notably present in the cecum, which might reflect the microbiota’s role in modulating host metabolic and addictive behaviors (Figure 8I). The transcriptional functions were predicted to be highly active in the cecum, suggesting a dynamic regulation of gene expression in these segments (Figure 8J).

## Discussion

The gut of animals is a highly complex and diverse microbial symbiotic environment, which forms a mutually beneficial relationship with the host (Costello et al., 2009; Barreto and Gordo, 2023). This relationship not only contributes to the normal development of the host but also plays a crucial role in maintaining its health, especially in preventing the imbalance of the intestinal microbial ecosystem (Alberdi et al., 2016; Ma et al., 2024). In short, these microorganisms and the host co-constructed a harmonious ecosystem vital for the host’s health (Rooks and Garrett, 2016). Therefore, maintaining intestinal microbial community diversity is very important for animal life activities. Due to the scarcity of studies on the intestinal microbiota of donkeys, this study characterized the gut microbiome of six healthy donkeys using high-throughput sequencing technology, aiming to investigate the abundance and potential functions of the microbiota at different locations in the gut.

The mammalian gut is compose of several different microenvironments, such as the duodenum, jejunum, ileum, cecum, and colon, which selectively housed microbial communities with specific characteristics along the longitudinal length of the intestinal cavity (Tropini et al., 2017). The diversity and abundance index change, which measures the stability of the intestinal microecological environment, is a crucial marker of host health and metabolic capacity (Li et al., 2018). Except for the Shannon index for the cecum, the Shannon index, Chao 1, and ACE were all higher in the mucosa samples than in the digested samples, as reported by Zhang et al. (2020). However, this study showed that alpha diversity (ACE, Chao, Shannon, Simpson, and Sobs) did not differ significantly between different intestinal sections. The results from the PCoA and the AMOVA analyses confirmed significant differences in the flora of different intestinal sites and fecal microbiome. The study revealed a substantial separation among the duodenum, jejunum, ileum, cecum, colon, and feces. These changes were hypothesized to be attributed to fundamental changes in feed. This external factor significantly altered the gut microbiome of donkeys (Lindenberg et al., 2019). The above results demonstrated that microbiota colonization was dynamic, influenced by external factors and interaction with the host, so the microbial community became more diverse until it reached a relatively balanced state (Pan and Yu, 2014; Ericsson et al., 2016; Zhou et al., 2021).

Regarding the richness and diversity of gut microbes, a comprehensive analysis was conducted using a Venn diagram. Only 339 bacterial species were identified across the six sites, most of which were endemic to their respective intestinal segments. In the current study, Shirazi-Beechey (2008) found that Firmicutes (69.21%) and Verrucomicrobia (18.13%) were the central bacterial communities in the intestinal microorganisms of horses, while Bacteroidetes, Proteobacteria, and Spirochaetes formed the bacterial communities to a lesser extent. Costa et al. (2012) reported that Firmicutes predominated (68%) among healthy horses, followed by Bacteroidetes (14%) and Proteobacteria (10%). The findings of this study indicated that the intestinal microbiota was mainly composed of Firmicutes, Bacteroides, and Verrucomicrobia. Yang et al. (2023) discovered that the intestinal microbiota of donkeys is primarily composed of Firmicutes and Bacteroidetes (>70%). Interestingly, these results were similar to those of previous studies. The donkey gut microbiota was found to be dominated by Firmicutes, Bacteroidetes, and Verrucomicrobia, followed by Patescibacteria, Spirochaetes, Actinobacteriota, and Proteobacteria. It has been reported that Firmicutes is the main microbial phylum promoting fiber decomposition in the gastrointestinal tract of herbivores (Dearing and Kohl, 2017), whose dominance may be related to the species’ anatomical physiology and feeding habits. These species ingest mainly insoluble fiber, using the cecum and large colon as the primary sites for fermentation (Costa et al., 2012). Concurrently, Firmicutes were mainly enriched in the cecum in this study. Bacteroidetes are the primary microbial phylum metabolizing carbohydrates in herbivores (Spence et al., 2006). Studies have shown that Bacteroidetes are involved in the normal development of the gastrointestinal tract (Thomas et al., 2011) and are the second largest intestinal microbiota in donkeys. Gut microbes play an essential role in carbohydrate metabolism because they produce numerous enzymes, including those involved in carbohydrate metabolism. Specifically, specific gut microbiota, such as Bacteroides and Prevotella (Aakko et al., 2020), affect the abundance of carbohydrate-metabolizing enzymes. The presence of these bacteria can promote efficient carbohydrate metabolism. Bacteroides play a vital role in the digestive system of herbivores, helping to break down carbohydrates and provide more nutrients for the host to absorb and utilize (Stappenbeck et al., 2002; Bäckhed et al., 2004). Additionally, the presence of Bacteroides also helps promote immune system development in herbivores, thereby enhancing the immune function of the host. In the gut, Firmicutes, Bacteroidetes, and Verrucomicrobia ferment dietary fiber to produce short-chain fatty acids (SCFAs), namely, butyric acid, propionic acid, and acetic acid. Verrucomicrobia exist in the inner lining of the intestinal mucosa and are abundant in healthy individuals. They can break down polysaccharides, such as mucopolysaccharides and cellulose, to provide energy and nutrients. These species appears to have an essential role in the cycling of high molecular weight compounds, showing an abundance of genes involved in the degradation of carbohydrates, especially sulfated polysaccharides (Herlemann et al., 2013; Landry et al., 2018; Bar-Shalom et al., 2023).

In this study, when the bacterial composition was examined at the genus level, *unclassified_f__Lachnospiraceae*, *Akkermansia*, *norank_f__F082*, *Christensenellaceae_R-7_group*, *Streptococcus*, *Rikenellaceae_RC9_gut_group*, *NK4A214_group*, and *UCG-005* emerged as the predominant genera. The genus *Lachnospiraceae*, belonging to Firmicutes, was identified as a potentially beneficial bacterium involved in the metabolism of various carbohydrates and ferments, leading to the production of acetic acid and butyric acid, the primary energy sources for the host. Several previous studies reported an association between *Lachnospiraceae* and the risk of T2D (Vacca et al., 2020; Ibrahim et al., 2021). SCFA pathways, including propylene glycol and acrylate signaling pathways, played an essential role in regulating the effects of *Lachnospiraceae* on T2D (O’Toole and Jeffery, 2015). A previous study showed that the proportion of *Akkermansia* species was highest in donkey fecal microbiota (Liu et al., 2014). *Akkermansia*, an intestinal bacterium that grows from gastrointestinal mucin, was closely related to immune response, lipid metabolism, and other bodily processes, playing a vital role in maintaining health. Meanwhile, *Lactobacillus*, identified as a genus of differential bacteria, was reported to possess non-fibrous carbohydrate-degrading capacities (e.g., pentoses, hexoses, and starch), thereby promoting cross-talk between the intestinal microbiota and the host and maintaining intestinal health. In summary, it could be inferred that the patterns of gut microbial diversity differed across the intestinal segments in this study. Different intestinal segments had apparent differences in function and metabolic bias. In summary, the PICRUSt functional prediction analysis of the delineated ten functional species revealed distinct capabilities across various intestinal segments in the equine gastrointestinal tract. Notably, the cecum demonstrated enhanced metabolic capacities in carbohydrates, prokaryotic cellular communities, antimicrobial drug resistance, membrane transport, signal transduction, and transcription when juxtaposed with the small intestine, which encompasses the jejunum and ileum. This suggested that a higher abundance of gut bacteria in the cecum was associated with the breaking down of carbohydrates. Furthermore, a comparative analysis between the large intestine (including the cecum and colon) and feces indicated a more pronounced functional activity in the cecum. Intriguingly, strong predictions were found in the cecum only for membrane transport and cellular community functions. This highlighted the fecal microbiome as a reservoir of a broader spectrum of microbial functions compared to the intestinal contents. However, the underlying mechanisms still need further exploration, particularly the association between colonization differences and metabolic function differences in different intestinal segments of the Dezhou donkeys. Understanding this relationship is crucial for improving intestinal development and growth performance in donkeys through nutritional conditioning.

## Conclusion

**Figure 9:**
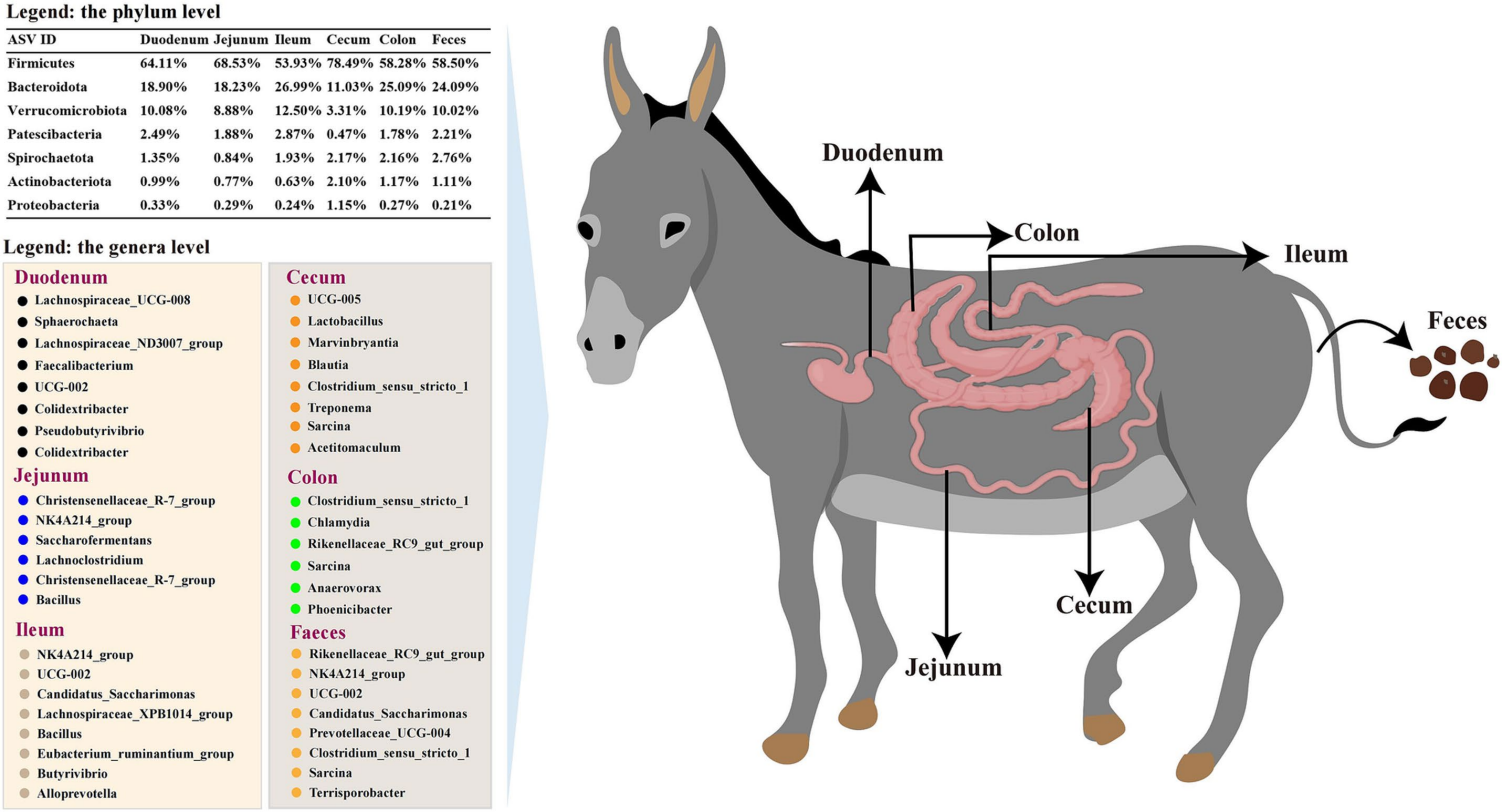
Summary of gut microbiota across the gastrointestinal tract of donkeys. The comprehensive analysis of the gut microbiota in donkeys revealed distinct microbial communities across various segments of the gastrointestinal tract, including the duodenum, jejunum, ileum, cecum, colon, and feces. The summary figure encapsulates the diversity at the genus and phylum levels, highlighting the unique microbial signatures of each segment.

This research showed that microbial communities in the donkey intestinal tract were abundant in diversity and population. Specific genera were significantly enriched in various segments: *Lachnospiraceae_UCG-008* and *Sphaerochaeta* in the duodenum; *Christensenellaceae_R-7_group* and *Bacillus* in the jejunum; *NK4A214_group* and *Alloprevotella* in the ileum; UCG-005 and *Lactobacillus* in the cecum; *Clostridium_sensu_stricto_1* and *Chlamydia* in the colon; *Rikenellaceae_RC9_gut_group* and *Prevotellaceae_UCG-004* in the feces (Figure 9). Moreover, PICRUSt2 functional prediction analysis indicated that carbohydrate metabolism, prokaryotic cellular communities, antimicrobial drug resistance, immune diseases, membrane transport, and signal transduction exhibited significant differences among the different intestinal segments, providing a deeper understanding of donkey gut microbes and clarifying the host gut function in donkeys to maintain body health.

## Data Availability

The data presented in the study are deposited in the NCBI repository, accession number PRJNA1204392.

## Footnotes

^1^ http://www.dongeejiao.com/

^2^ http://www.r-project.org/

^3^ https://github.com/picrust/picrust2
